# Genome-wide identification of starch phosphorylase gene family in *Rosa chinensis* and expression in response to abiotic stress

**DOI:** 10.1038/s41598-024-64937-1

**Published:** 2024-06-17

**Authors:** Xu Li, Yang Chen, Zaiqi Zhang, Qin He, Tingting Tian, Yangmiao Jiao, Liang Cao

**Affiliations:** Hunan Provincial Key Laboratory of Dong Medicine, Ethnic Medicine Research Center, Hunan University of Medicine, Huaihua, 418000 China

**Keywords:** *Rosa chinensis*, Starch phosphorylase, Stress treatment, Evolution, Agricultural genetics, Evolutionary biology, Gene expression, Plant genetics

## Abstract

Chinese rose (*Rosa chinensis*) is an important ornamental plant, with economic, cultural, and symbolic significance. During the application of outdoor greening, adverse environments such as high temperature and drought are often encountered, which affect its application scope and ornamental quality. The starch phosphorylase (*Pho*) gene family participate in the synthesis and decomposition of starch, not only related to plant energy metabolism, but also plays an important role in plant stress resistance. The role of *Pho* in combating salinity and high temperature stress in *R. chinensis* remains unknown. In this work, 4 *Phos* from *R. chinensis* were detected with Pfam number of Pho (PF00343.23) and predicted by homolog-based prediction (HBP). The *Phos* are characterized by sequence lengths of 821 to 997 bp, and the proteins are predicted to subcellularly located in the plastid and cytoplasm. The regulatory regions of the *Phos* contain abundant stress and phytohormone-responsive cis-acting elements. Based on transcriptome analysis, the *Phos* were found to respond to abiotic stress factors such as drought, salinity, high temperature, and plant phytohormone of jasmonic acid and salicylic acid. The response of *Phos* to abiotic stress factors such as salinity and high temperature was confirmed by qRT-PCR analysis. To evaluate the genetic characteristics of *Phos*, a total of 69 *Phos* from 17 species were analyzed and then classified into 3 groups in phylogenetic tree. The collinearity analysis of *Phos* in *R. chinensis* and other species was conducted for the first time. This work provides a view of evolution for the *Pho* gene family and indicates that *Phos* play an important role in abiotic stress response of *R. chinensis*.

## Introduction

Plants are subjected to various biotic and abiotic factors during the growth and reproduction phases. Stress beyond the normal range usually has adverse effects on plant physiology, including the production of reactive oxygen species (ROS), decreased membrane stability, protein structural degeneration, and metabolic disorders. In order to cope with stress factors, plants have developed various adaptation mechanisms^1^. Starch is a major component of many grains and feeds, and also an important industrial raw material. More than 60 biochemical reactions and complex physiological regulation are involved in the synthesis and accumulation of starch, which also plays an important role in plant growth, development, and stress responses^2^. Drought treatment significantly induced gene transcription involved in the metabolism of starch and sugars (fructose and mannose)^3^. The sugars produced by starch degradation can serve as osmo-regulation substances, improving the ability of cells to absorb water.

Starch phosphorylase (*Pho*) is a member of the GT35-glycogen-phosphorylase superfamily^4^. It plays a dynamic regulatory role in the synthesis and degradation of starch, catalyzing the synthesis of glucose 1-P (G1P) to glucan or catalyzing *α*-glucan phosphorylation^5^. Two types of starch phosphorylase are found in higher plants: plastidial starch phosphorylase (Pho1) and cytosolic starch phosphorylase (Pho2)^6^. Those two forms have significant sequence similarities with each other, but they differ in molecular size, substrate specificity, physiological effects, and intracellular localization^7^. Starch phosphorylase has been widely studied in *Arabidopsis thaliana*^8,9^, *Oryza sativa*^10^, *Zea mays*^11^, wheat (*Triticum aestivum* L.)^12^, and potato (*Solanum tuberosum* L.)^13^. In *A. thaliana*, *AtPho*1 seems to play a role in abiotic stress rather than in starch degradation^8^. The role of *Pho* in starch synthesis and decomposition has been widely researched, while the gene family’s action in stress resistance via carbohydrate metabolism is worth further research^8,14^.

Roses (*Rosa chinensis*) is an important ornamental plant, with economic, cultural, and symbolic significance^15^. They are used not only as fresh cut flowers but also widely used as outdoor landscaping flowers^16^. During the application of outdoor greening, adverse environments such as high temperature and drought are often encountered, which affect its application scope and ornamental quality^17^. In the study of drought stress in roses, it was found out that with the deepening of drought, the content of proline and soluble sugar increased^18^. Phytohormone signal transduction was also a pathway in regulating the salt tolerance response of *Rosa chinensis*^19^. *Phos* play an important role in the synthesis and decomposition of starch, not only related to plant energy metabolism, but also to plant stress resistance^8,14^. The role of *Phos* in combating high temperature and drought treatment in *R. chinensis* is unclear. Thus, we identified the *Phos* family in *R. chinensis* and analyzed the expression levels of these genes under different stress treatments. This work provides new insights on the *Pho* gene family response to abiotic stress.

## Materials and methods

### Identification of *Pho* gene family

Fourteen genomes were obtained from public genetic data services (Table 1), for detailed information, see Supplementary Table S1. The HMMER (v3.2)^20^ and Pfam number of Pho (PF00343.23) were used to search Pho protein sequence (E-value < 1e^−10^). In order to minimize the deviation caused by different gene annotation methods, we used homolog-based prediction (HBP) as a supplement tool to predict genes (Supplementary Table S2)^21^. Genes with short annotation were merged along with adjustments of the promoter region. The resulted genes were tested for the functional annotation by KEGG (https://www.genome.jp/kegg/) and Swissprot (release-2021_03) (http://www.ebi.ac.uk/sprot). The motif was recognized using Meme (https://meme-suite.org/meme/tools/meme), with a number of 11^22^. The domain was predicted using CDD (https://www.ncbi.nlm.nih.gov/cdd/) database. Enzyme function prediction was performed using CLEAN software (https://clean.platform.moleculemaker.org/configuration)^23^.

**Table 1:** The genomes information of 17 species and 69 *Phos* identified.

Species	Origin of the genome Specific version/Release date	*Phos* number	References
*Rosa chinensis* Old Blush	Rosa_chinensis_Old_Blush_homozygous_genome-v2.0.fna.gz	4	^24^
*Fragaria vesca*	Fragaria_vesca_v4.0.a1.fasta.gz (accessed on 1 January 2018)	4	^25^
*Rosa rugosa*	Rosa_rugosa_genome.fasta.gz (accessed on 31 August 2021)	4	^26^
*Rubus chingii* Hu	Rubus_chingii_Hu_fina.fa.gz	4	^27^
*Malus x domestica* HFTH1	Malus_x_domestica_HFTH1_v1.0.a1/assembly/HFTH1.all.chr.fa.gz	6	^28^
*Pyrus pyrifolia*	Pyrus_pyrifolia/ppyrifolia_v1.0/assembly/PPY_r1.0.pmol.fasta.gz (accessed on 11 January 2021)	6	^29^
*Prunus persica*	Prunus_persica/Ppersica_Zhongyoutao14_v1.0/assembly/CN14.genome.fa.gz	4	^30^
*Crataegus pinnatifida*	Crataegus_pinnatifida/hawthorn_v1/assembly/hawthorn.LG.fasta.gz	6	^31^
*Ziziphus jujuba*	GCF_020796205.1_ASM2079620v1_genomic.fna.gz	3	^32^
*Vitis vinifera*	GCF_000003745.3_12X_genomic.fna.gz (accessed on 7 November 2019)	4	^33^
*Medicago truncatula*	GCF_003473485.1_MtrunA17r5.0-ANR_genomic.fna.gz	4	^34^
Cannabis sativa	GCF_900626175.2_cs10/GCF_900626175.2_cs10_genomic.fna.gz	3	^35^
*Malus prunifolia* Fupingqiuzi	Malus_prunifolia_Fupingqiuzi.genome_sequence.fasta.gz	7	^36^
*Potentilla micrantha*	Potentilla_draft_genome.fasta.gz (accessed on 4 May 2018)	4	^37^
*Oryza sativa*	GCF_001433935.1_IRGSP-1.0_genomic.fna.gz	2	^10,38^
*Arabidopsis thaliana*	GCF_000001735.4_TAIR10.1_genomic.fna.gz	2	^8,9^
Maize_B73_V5	GCF_902167145.1_Zm-B73-REFERENCE-NAM-5.0_genomic.fna.gz	2	^11^

### Sequence alignment and phylogenetic analysis

Protein sequence alignments were conducted by muscle 5.1^39^. The phylogenetic tree was then constructed using iqtree (v1.5.5) under the MFP model^40^, using maximum likelihood with parameters of 1000 replicates of bootstrap, the results were displayed by the Interactive Tree of Life (itol), an online tool for the display, annotation and management of phylogenetic trees (https://itol.embl.de/upload.cgi).

### Physicochemical properties and protein structure prediction of Pho in *R. chinensis*

Database of ExPASy (http://expasy.org/protparam/)^41^ was used to predict the number of amino acids, sequence coding for amino acids in protein (CDS), isoelectric point (Acidity < 7, alkalinity > 7), molecular weight, instability index (stable < 40), aliphatic index (hydrophilic < 100), and grand average of hydropathicity (GRAVY) (hydrophilic < 0). The subcellular localization was predicted using BUSCA (http://busca.biocomp.unibo.it/)^42^. The three-dimensional (3D) structures of Pho proteins were predicted by websever of Phyre2 (protein homology/analogy recognition engine V 2.0)^43^.

### Prediction of promoter *cis*-acting elements

2000 bp sequence upstream of *Pho* was extracted, and the cis-acting elements were predicted and analyzed by PlantCARE (http://bioinformatics.psb.ugent.be/webtools/plantcare/html/)^44^. The website analyzed the cis-acting elements within the promoter region, such as hormone regulatory elements, growth and development related regulatory elements, or stress regulatory elements.

### Collinearity and gene positive selection analysis

Collinearity of *Phos* in *R. chinensis*, *R. rugosa*, *R. chingii* and *F. vesca* were analyzed by using the tool of jcvi (v1.1.12) (https://www.jcvi.org/)^45^ under parameters of cscore = 0.99 and minspan = 30. Tbtools (v1.120)^46^ was applied to determine the collinearity of *Vitis vinifera*, *Ziziphus jujuba*, *R. chinensis* with parameter of e < 10^–5^. Average nucleotide identity (ANI) estimation of 12 genomes was carried out by FastANI (v1.31)^47^. The gene positive selection analysis of dN/dS was performed by paml (v4.8)^48^.

### Transcriptome analysis of *Phos* under stress treatment

Transcriptome data of roses under different stress treatments were downloaded from the NCBI Sequence Read Archive (SRA) database (Supplementary Table S3), including gradual drought stress (PRJNA722055)^3^, salt stress (PRJNA689657), heat stress (PRJNA473465)^49^ and phytohormones stress (PRJNA522664)^50^. The mentioned transcriptome data was filtered using Trimmomatic (v0.38)^51^ with the parameters setting of (Headcrop:2, Leading:20, trailing:20, Sliding window:5:20, Minlen:50, Avgqual:20). Transcriptome analysis of gene expression by RNA sequencing (RNA-seq) using the HISAT2 (v2.1.0)-Stringtie (v1.3.4d)-DESeq2 (v1.30.1) RNASeq pipeline^52^. The parameters of DESeq2 (v1.30.1) analysis are absolute fold change ≥ 2, false discovery rate < 0.05^53^.

### qRT-PCR Analysis of *Phos* response to high temperature and salt

Two-year-old cutting seedlings of *R. chinensis* used in the experiments were purchased from Huaihua flower market (109.938318° E, 27.410230° N) and identified by Prof. Liang Cao. The plant materials, nearly identical in size, were planted in the pots (20 cm in diameter, 35 cm in height) filled with mixed sterilized soil of nutritive soil, garden soil, perlite, and vermiculite in the ratio of 5:3:1:1 (v/v). Before high temperature and salt treatment, all samples were cultured under conditions of 70% relative humidity and day/night temperatures of 25 °C/20 °C in the same artificial climate chamber at Hunan University of Medicine. Subsequently, the samples were subjected to a high temperature (45 °C) and salt (0.4%, NaCl: Na_2_SO_4_ = 1:2 in molar) treatment for 2 h. Each treatment group consisted of three biological replicates. Total RNA was extracted from the fourth or fifth fully expanded leaves in control (CK), high temperature and slat treatment, using an OminiPlant RNA Kit (CWBIO., Jiangsu, China) and a PrimeScript™ RT reagent Kit with gDNA Eraser (TransGen Biotech, Beijing, China) for reverse transcription synthesis of complementary DNA, following the manufacturer’s instructions. Four *Phos* from *R. chinensis* were selected for qRT-PCR analysis. GAPDH was used as the reference gene, and the primer sequences are presented in Supplementary Table S4. The fluorescence signals were detected using a LightCycle 480 Real-Time System (Roche Diagnostics Ltd., Rotkreuz, Switzerland) after configuring the PCR reaction had with PerfectStart Green qPCR SuperMix (TransGen Biotech, Beijing, China). For each biological replicate, three technical replicates were performed, respectively.

### Data analysis

The primary outcomes will be compared between the groups using a *t*-Test in SPSS (Statistical Package for the Social Sciences) software, and an analysis of variance for repeated measurements will be used for data analysis.

### Plant guideline statement

Experimental research and field studies on plants (either cultivated or wild), including the collection of plant material, comply with relevant institutional, national, and international guidelines and legislation.

## Results

### Identification of *Pho* gene family

Sixty-three *Phos* were retrieved from fourteen genomes, namely *V. Vinifera*, *M. truncatula*, *Z. Jujuba*, *C. sativa* and ten species from Rosaceae family including *R. chinensis*. Among them, the short annotations of *MP16G1059300* and *MP16G1059400* merged into *MP16G1059300m*, *Cpi13g547* and *Cpi13g548* merged into *Cpi13g547m* by using HBP analysis. The number of *Phos* for *V. vinifera*, *M. Truncatula*, *Z. Jujuba*, *C. sativa* was 4, 4, 3, 3, respectively. For *F. vesca*, *R. chingii*, *P. micrantha*, *R. rugosa* and *R. chinensis*, four *Phos* were detected from each of them. In genome of *R. chinensis*, *Rco2g0166331* is located at the terminal of chromosome 2, *Rco4g0438671* located on chromosome 4, while *Rco5g0009141* and *Rco5g0068321* are located on chromosome 5. A number of 6, 4, 6, 6, and 7 *Phos* were obtained for *C. pinnatifida*, *P. persica*, *P. pyrifolia*, *Malus x domestica*, *M. prunifolia*, respectively (Supplementary Table S1).

### Phylogenetic analysis

**Figure 1:**
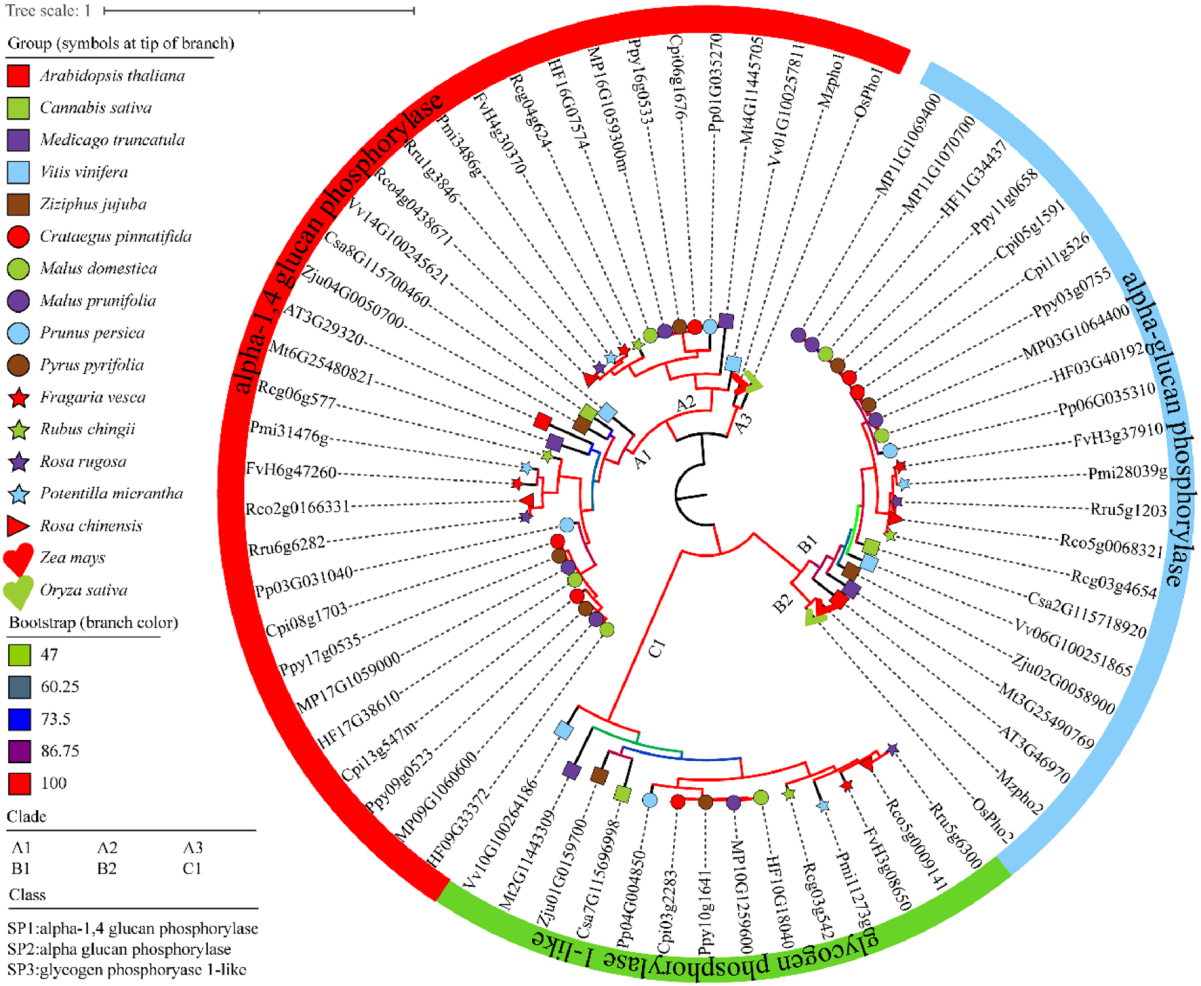
Phylogenetic tree of 69 *Phos* retrieved from 17 species of *V. vinifera* (Vvi), *M. truncatula* (Mtr), *Z. jujuba* (Zju), *C. sativa* (Csa), *F. vesca* (Fve), *R. chingii* (Rcg), *P. micrantha* (Pmi), *R. rugosa* (Rru), *R. chinensis* (Rco), *C. pinnatifida* (Cpi), *P. persica* (Ppe), *P. pyrifolia* (Ppy), *Malus × domestica* (Mdo), *M. prunifolia* (Mpr), *A. thaliana* (Ath), *O. sativa* (Osa) and *Z. mays* (Zma).

Phylogenetic tree was constructed by combining the published 6 *Phos* from *A thaliana*, *O. sativa* and *Z. mays* (Fig. 1, Table 1), in total, 69 *Phos* from 17 species were classified into three groups, which fit with the functional annotation of SP1 (alpha-1,4 glucan phosphorylase), SP2 (alpha-glucan phosphorylase) and SP3 (glycogen phosphorylase 1-like). The group of SP1 contains three subgroup, namely clades A1 to A3. In clade of A1, *V. vinifera*, *A. thaliana*, and nine species of rosids were single copy, while *C. pinnatifida*, *P. Pyrifolia*, *Malus × domestica* and *M. prunifolia* were double copies. In A2 clade, twelve species with single copy were gathered. Single copy form *O. sativa* and *Z. mays* were classified in clade A3. SP2 contains two clades of B1 and B2, in clade B1, *V. vinifera*, *A. thaliana*, and nine species of rosids were single copy, while for species of *C. pinnatifida*, *P. pyrifolia*, *Malus × domestica*, two copies for each of them, and three copies for *M. prunifolia*. In clade B2 contains single copy for each of *O. sativa* and *Z. mays*. In the SP3 clade, *V. vinifera* and thirteen other species were single copy.

### Physicochemical properties of Pho in *R. chinensis*

**Figure 2:**
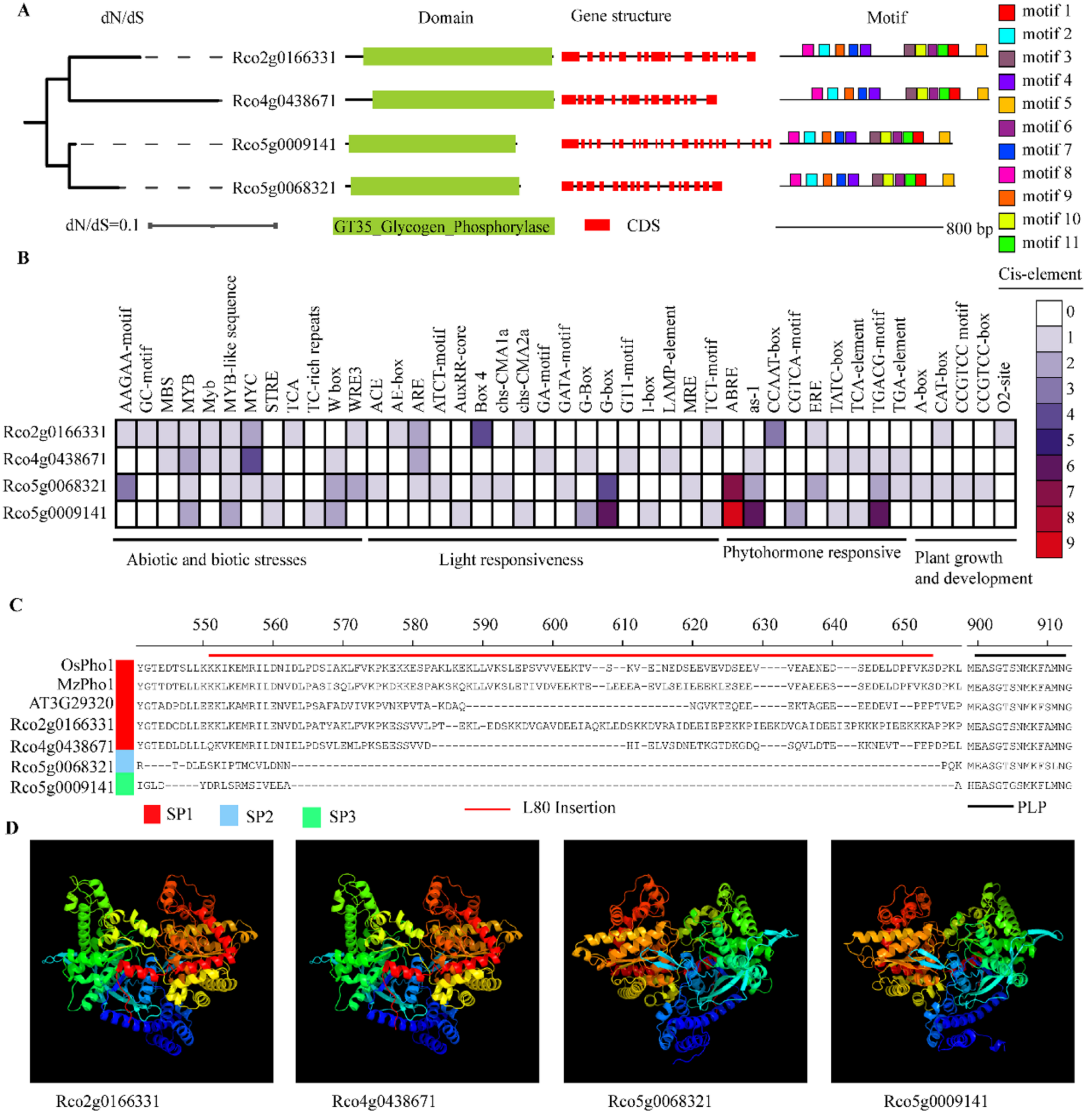
Physicochemical properties and structure of four *Phos* in *R. Chinensis* (Rco), namely *Rco2g0166331*, *Rco4g0438671*, *Rco5g0068321*, *Rco5g0009141*. (**A**) Gene structure (dN/Ds, domain, CDS, and motif) of *Phos* identified from *R. Chinensis* (Rco). (**B**) Cis-acting element identified from 2.0 kb upstream of the *Phos*. (**C**) Sequence alignment of L80 insertion and PLP (phosphorylase pyridoxal-phosphate) attachment site in four Phos of *R. Chinensis* and three model plants of *O. sativa* (OsPho1), *A. thaliana* (AT3G29320), *Z. mays* (MzPho1). (**D**) The three-dimensional (3D) structures of Pho proteins predicted by Phyre2.

Four copies of *Phos* were screened from *R. chinensis* in this work, *Rco2g0166331*, *Rco4g0438671*, *Rco5g0009141*, and *Rco5g0068321*, with lengths ranging from 821 to 997 base pairs and 14 to 22 CDSs. The calculated theoretical pI < 7, aliphatic index < 100, and GRAVY < 0, indicate weak acidity and hydrophilicity. In the subcellular localization analysis, *Rco2g0166331* was deduced to be located in the mitochondrion, *Rco4g0438671* in the chloroplast, and *Rco5g0009141* and *Rco5g0068321* were assumed to be expressed in the cytoplasm (Supplementary Table S5). The calculation of dN/dS (ω < 1) indicates negative selection, and eleven common motifs of these four *Phos* were identified (Fig. 2A), and the GT35_Glycogen_Phosphorylase domain was contained in the proteins, showing high consistency in sequences (Fig. 2A; Supplementary Table S6).

### Promoter *cis*-acting elements and protein structure analysis of *Phos* in *R. chinensis*

In order to investigate the biological functions of the *Pho* gene family in the growth, development, and stress resistance of *R. chinensis*, we analyzed the 2.0 kb sequence upstream of the *Phos* and identified cis-acting elements consistent with the gene transcription direction (Fig. 2B). All predicted cis-acting elements were classified into 4 types: abiotic and biotic stress, light response, phytohormone response, and plant growth and development (Fig. 2B). MYB binding site (MBS) (drought-inducibility) were detected in *Rco2g0166331* and *Rco4g0438671*. The stress-responsive element (STRE, AGGGG) for heat and osmotic stress were detected in *Rco5g0009141* and *Rco5g0068321*. The low/hypoxia stress element GC motif and low temperature response element TCA were only present in *Rco2g0166331*. TC rich repeats for defense were only found in *Rco5g0009141*, and MYB and MYB-like sequences were detected in all four *Phos*. ACE, ARE, Box 4, G-Box and GT1-motif and other light response elements were widely distributed among the four *Phos*. In the phytohormone-responsive cis-acting elements, we detected abscisic acid-responsive elements (ABRE) responsive to abscisic acid, as-1 and TCA-element responsive to salicylic acid, and CCAAT-box responsive to ethylene synthesis. CGTCA-motif and TGACG-motif involved in the MeJA-responsiveness, estrogen response element (ERE) involved in the estrogen-response, TATC-box involved in the gibberellin-responsiveness, and TGA-element involved in auxin responsiveness were also detected. Five cis-acting elements: A-box, CAT-box, CCGTCC motif, CCGTCC-box, and O_2_-site, which participate in plant growth and development were screened. Among these, the CAT box and CCGTCC motif are cis-acting elements related to meristem-specific activation.

The functional domains of Pho were aligned, then the protein structure of *Phos* were predicted (Fig. 2C,D), revealing that SP1 differrs from SP2 and SP3 by an L80 insertion. There is also a significant difference in the L80 insertion domain between monocots and dicots, as well as a significant variation between *R. chinensis* and *A. thaliana* (Fig. 2C). The PLP is phosphorylase pyridoxal-phosphate attachment site, all known phosphorylases share the catalytic and structural properties, and the sequence is highly conserved. We discovered that *Rco2g0166331*and *Rco4g0438671* shared the same PLP sequence with *OsPho1* and *MzPho1*, while *Rco5g0068321* and *Rco5g0009141* have variations of 2 and 4 amino acids, respectively (Fig. 2C). The variation in genetic sequences resulted in different three-dimensional (3D) protein structures, with two copies of SP1(*Rco2g0166331*, *Rco4g0438671*) showing similar structures, distinct from those of SP2 (*Rco5g0068321*) and SP3 (*Rco5g0009141*) (Fig. 2D).

### Average nucleotide identity and intergenomic collinearity of *Pho* gene family

**Figure 3:**
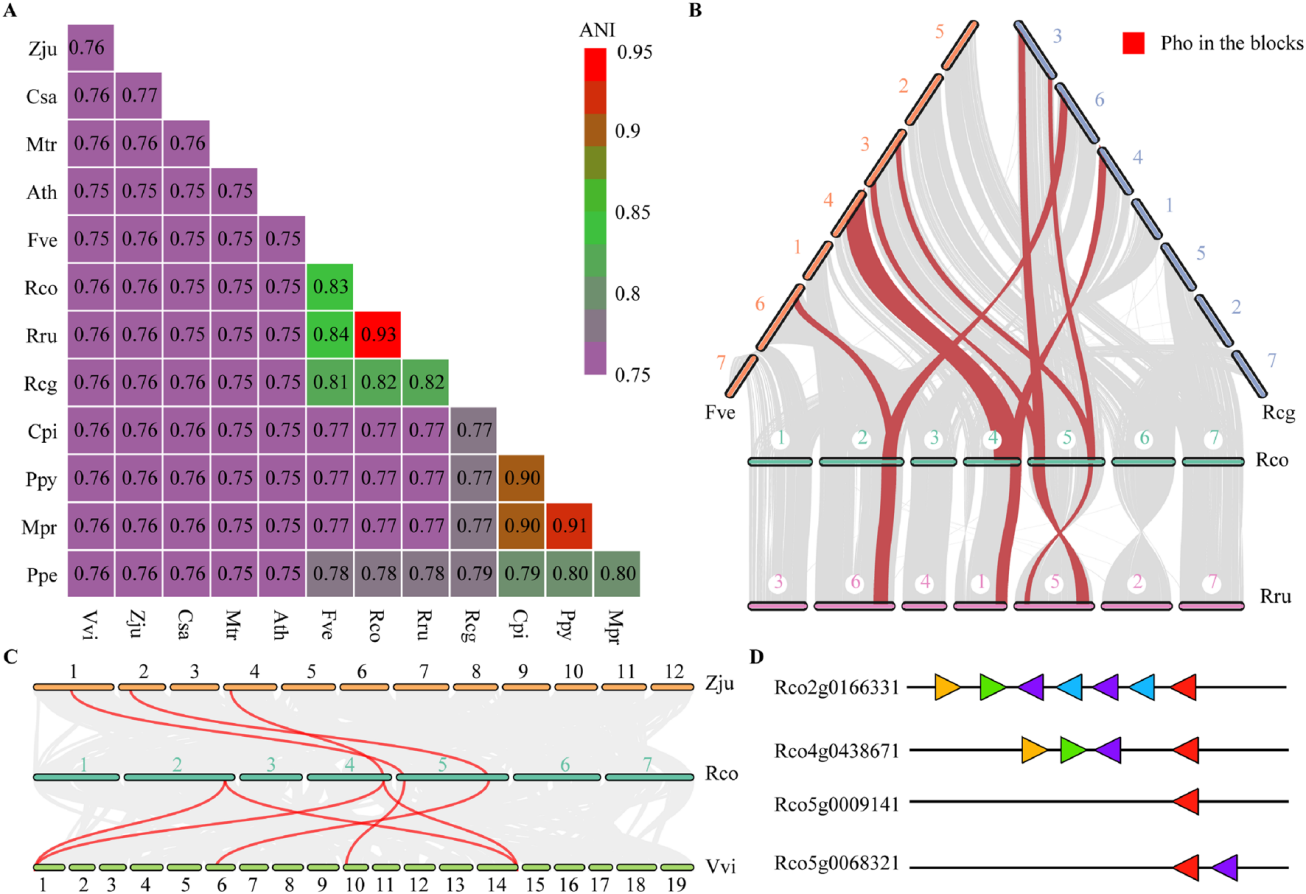
ANI and collinearity analysis of *Pho* gene family. (**A**) ANI in 12 species [Abbreviation: *V. vinifera* (Vvi), *M. truncatula* (Mtr), *Z. jujuba* (Zju), *C. sativa* (Csa), *F. vesca* (Fve), *R. chingii* (Rcg), *R. rugosa* (Rru), *R. chinensis* (Rco), *C. pinnatifida* (Cpi), *P. persica* (Ppe), *P. pyrifolia* (Ppy) , *M. prunifolia* (Mpr), *A. thaliana* (Ath)]. (**B**) Collinearity analysis of *Phos* in Rco, Rru, Rcg and Fve. (**C**) Collinearity analysis of *Phos* in Rco, Vvi and Zju (the red line represents *Pho*). (**D**) Homology of 6 genes both upstream and downstream of the *Pho* gene family (the same color triangle represents homologous genes, the direction of triangle represents gene transcription direction, the red triangle represents *Pho*).

Average nucleotide identity (ANI) between *R. chinensis* and eleven other genomes were summarized (Fig. 3A). *R. chinensis* have close ANI of 0.93 to *R. rugosa*, as they are from the same genus. The ANI value in 0.75–0.83 between *R. chinensis* and other species. *P. pyrifolia* and *C. pinnatifida*, *M. prunifolia* and *C. pinnatifida*, *M. prunifolia* and *P. pyrifolia* have ANI value of 0.90, 0.90, 0.91 respectively, means they are close in genetic background. Collinearity analysis of *Phos* in *R. chinensis*, *R. rugosa*, *R. chingii*, and *F. vesca* were conducted using jcvi (v1.1.12)^45^ software (Fig. 3B). The block where the *Phos* are located exhibits good collinearity among four species, and there is chromosomal segment inversion between *R. chinensis* and *R. rugosa*. Tbtools was used to investigate the collinearity relationship of *Phos* among the genomes of *R. chinensis*, *V. vinifera*, and *Z. jujuba* (Fig. 3C). *Rco2g0166331* and *Rco4g0438671* each have two homologous copies in the genome of *V. vinifera*, while *Rco2g0166331* have no homologous copy in *Z. Jujuba*. We analyzed the homology of 6 linkages genes both upstream and downstream of the *Pho* gene family (Fig. 3D). *Rco2g0166331* and *Rco4g0438671* have three homologous genes upstream, *Rco5g0068321* have one homologous gene downstream, while *Rco5g0009141* have no homologous gene.

### Expression patterns of *Pho* gene family in different stress treatment

**Figure 4:**
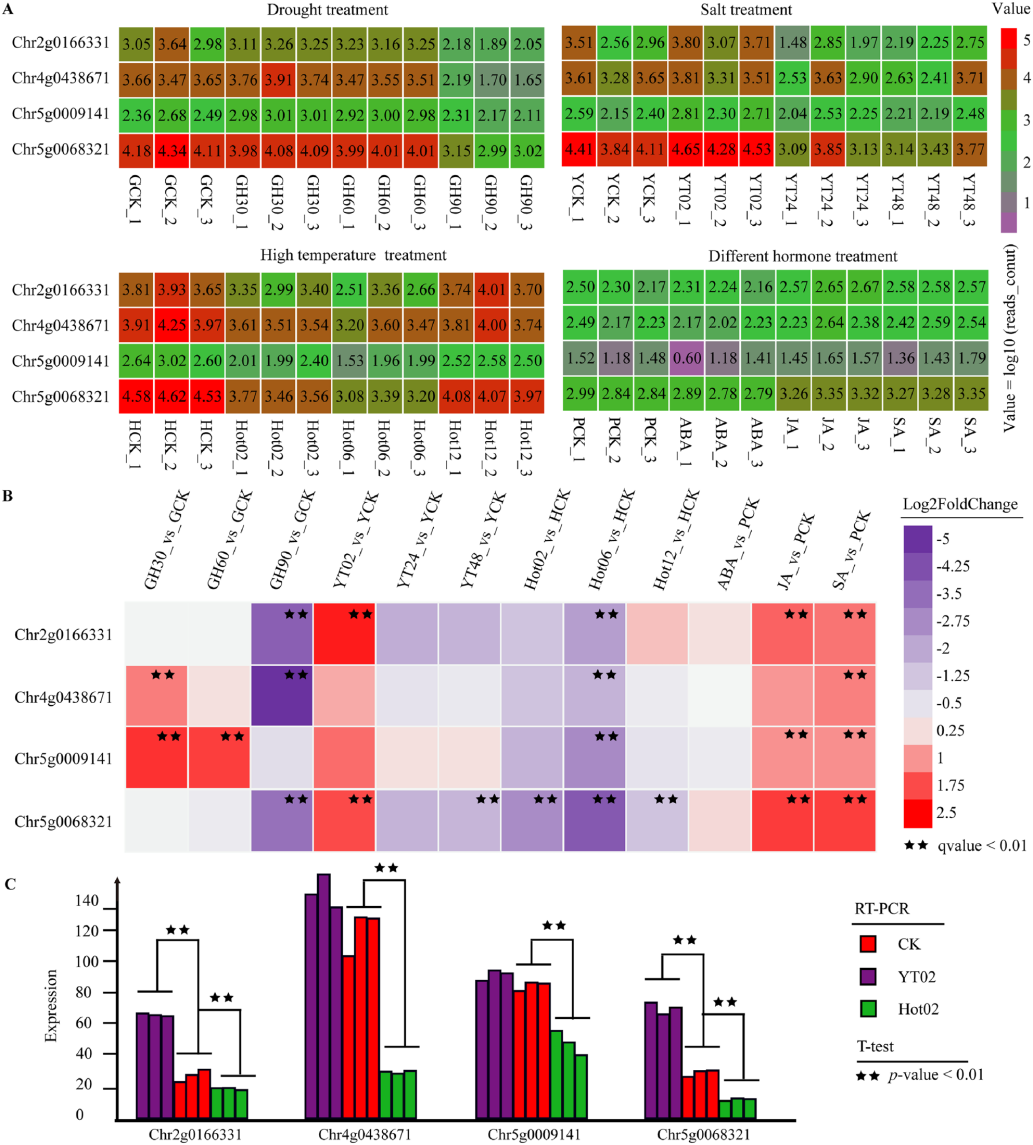
Expression patterns of the *Phos* in different stress treatment. (**A**) Heat map of 4 *Phos* under different stress treatment. (**B**) Statistic of expression level of 4 *Phos* under different stress treatment (GCK: control of drought treatment, GH30, GH60, GH90: drought treatment of 30 days, 60 days, and 90 days, respectively; YCK: control of salt treatment, YT02, YT24, YT48: salt treatment for 2 h, 24 h, and 48 h, respectively; HCK: control of high temperature treatment, Hot02, Hot06, Hot12: high temperature treatment for 2 h, 6 h and 12 h respectively; PCK: control of phytohormone treatment, ABA, JA, and SA represents abscisic acid, jasmonic acid, and salicylic acid treatment for 24 h respectively). (**C**) Transcription of *Phos* in hot and salt treatment.

To elucidate the expression patterns of the *Pho* gene family of *R. chinensis* under stress treatment, we analyzed its gene expression levels under conditions of drought, salinity, high temperature, and different phytohormone treatments (Fig. 4A). *Rco5g0009141* was significantly upregulated after 30 and 60 days of drought treatment, while *Rco4g0438671* was upregulated after 30 days of drought treatment (Fig. 4B). *Rco2g0166331*, *Rco4g0438671*, and *Rco5g0068321* were significantly downregulated after 90 days of drought treatment. After 2 h of salt treatment, the expression levels of *Rco2g0166331* and *Rco5g0068321* were significantly upregulated, whereas the expression levels of *Rco5g0068321* were significantly downregulated after 48 h of treatment. Under high temperature treatment (45 °C), *Rco5g0068321* was significantly downregulated at 2 h, 6 h, and 12 h, while *Rco2g0166331*, *Rco4g0438671*, and *Rco5g0009141* were significantly downregulated under 6 h of treatment. The expression levels of *Phos* under different phytohormones treatment for 24 h were analyzed. It was demonstrated that there was no significant change in four *Phos* under 100 μM abscisic acid (ABA) treatment. The expression levels of *Rco2g0166331*, *Rco5g0009141*, and *Rco5g0068321* were significantly up-regulated after 50 μM jasmonic acid (JA) treatment, and all four *Phos* were significantly up-regulated under 100 μM salicylic acid (SA) treatment.

To conform the transcription level of the *Phos*, high temperature and salt treatment were administered for two hours each. The four *Phos* were amplified by using qRT-PCR (Fig. 4C). Under salt treatment, *Rco2g0166331* and *Rco5g0068321* were significantly upregulated, consistent with the transcriptome data results. While four *Phos* were significantly downregulated under 2 h of high temperature treatment, only one gene (*Rco5g0068321*) was significantly downregulated in transcriptome data (the other 3 genes were also downregulated), indicating that all *Phos* respond to high temperature stress.

## Discussion

During the process of evolution, it is common for plants to undergo genome doubling or gene amplification events to adapt to environmental changes. The generated duplicate genes are an important source of genome evolution, and provide the foundation for regulation and function diversity^54,55^. Grape has not undergone polyploidization since the *γ* event^56^. Similarly, jujube has also not undergone WGD after the *γ* event^57^. On the other hand, pears and apples have undergone recent whole-genome duplication events^58^. All these species belong to the rosids. Based on the results of the phylogenetic tree (Fig. 1), we believe that the three groups SP1, SP2, and SP3 are the result of whole-genome triplication in rosids.

According to the phylogenetic tree, *Rco4g0438671* (SP1) and *Rco5g0068321* (SP2) are believed to be essential for maintaining metabolism. *Rco2g0166331* (SP1) may be redundant copy produced by the expansion or replication of *Rco4g0438671*, based on the collinearity (Fig. 3C) and gene linkage (Fig. 3D), with the expression levels of the two genes tending to be consistent under different treatments (Fig. 4A). *Rco5g0009141* (SP3) may be a copy derived from genome duplication^59^. Therefore, the upstream and downstream regions of *Rco5g0009141* do not share syntonic blocks with other genes, and sequence variation also observed due to long-term evolution. The expression level of *Rco5g0009141* was lower than that of *Rco5g0068321* under different stress treatments (Fig. 4A), and the latter is necessary for maintaining cellular metabolism. Therefore, *Rco5g0009141* can undergo relatively greater arbitrariness evolution.

Carbohydrates play an important role in maintaining plant’s physiology conditions and coping with abiotic stresses. *Rco4g0438671* and *Rco5g0009141* were significantly upregulated after 30 days of drought treatment, accelerating starch decomposition to improve drought resistance. After 90 days of drought treatment, the leaves severely curled and withered, *Rco2g0166331*, *Rco4g0438671*, and *Rco5g0068321* were significantly downregulated^3^. The expression levels of *Phos* were upregulated after 2 h of salt treatment, particularly *Rco2g0166331* and *Rco5g0068321*, which were significantly upregulated, participating in starch catabolism to maintain cellular osmotic balance. Under high temperature stress, the expression levels of four *Phos* were significantly downregulated at 6 h of treatment, and in qRT-PCR analysis, the transcription level were observed to be significantly downregulated as early as 2 h into the treatment, indicating the *Phos* response in high temperature in maintain the carbon balance and energy homeostasis^60^. Under treatment with 50 μM JA and 100 μM SA, the expression levels of four *Phos* were upregulated, indicating that these genes can respond to phytohormone signals e involved in plant growth and stress resistance. The cis-acting elements of as-1 and TCA-element, responsive to SA were detected in the gene structure, indicating the involvement *Phos* in the hormone-mediated stress resistance. Many light-responsive cis-elements also detected in the *Phos*, suggesting that the expression of these genes may be related to photosynthesis progression^17^. The *Phos* can respond to abiotic stress factors providing new insights into the stress resistance mechanisms of *R. chinensis*.

## Conclusions

The starch phosphorylase (*Pho*) genes are involved in the reversible process of starch synthesis and decomposition. In this work, 69 *Phos* from 17 species were gathered to construct the phylogenetic tree, the genes were classified by catalyze activity. The structure of *Phos* in *R. chinensis* were determined by using bioinformatic method. The expression levels of *Phos* under different stress treatment were analyzed using transcription database and qRT-PCR experiment. The results shown that *Phos* can respond to abiotic stress factors such as drought and salinity, as well as phytohormone signals. This work provides new insights into the role of *Phos* in stress resistance in *R. chinensis*.

### Supplementary Information

Supplementary Tables.

## Data Availability

Data are available from the corresponding authors on request.
